# BKPyVN-associated macrophage subpopulations and diagnostic biomarker identification - combine machine learning with single-cell analysis

**DOI:** 10.3389/fimmu.2026.1881621

**Published:** 2026-10-14

**Authors:** Shengxian Xu, Guodong Zhao, Yingzhen He, Junying Mo, Gang Huang

**Affiliations:** Organ Transplant Center, The First Affiliated Hospital of Sun Yat-Sen University, Guangzhou, China

**Keywords:** BKPyVN, hdWGCNA, machine learning, macrophages, prediction models, ScRNA-seq

## Abstract

**Background:**

As pivotal effector cells of innate immunity, macrophages may play a critical role in immune regulation and fibrotic remodeling in BK polyomavirus nephropathy (BKPyVN) following kidney transplantation. However, their subpopulation heterogeneity, molecular characteristics, and functional mechanisms remain poorly understood.

**Methods:**

We integrated single-cell RNA sequencing (scRNA-seq) data from BKPyVN and stable transplant kidney (STK) biopsies. Pseudotime trajectory, CellChat, and high-dimensional weighted gene co-expression network analysis (hdWGCNA) were used to characterize BKPyVN-associated macrophages (BKPyVN-Mac) and identify related gene modules. Characteristic genes from these modules were used to construct diagnostic models from an integrated renal allograft microarray dataset, and nine machine-learning algorithms were compared. Key genes were validated by RT-qPCR and immunohistochemistry in human biopsies and by time-course transcript and protein analyses in a persistent mouse polyomavirus (MPyV) infection model.

**Results:**

BKPyVN-Mac occupied a disease-associated macrophage state and may serve as a central communication hub within the allograft microenvironment. The support vector machine model exhibited the best diagnostic performance, with the highest area under the receiver operating characteristic curve (AUC = 0.923). ATF4 and MCL1 were identified as key feature genes, and their upregulation was confirmed in human BKPyVN biopsies and, through their mouse orthologs Atf4 and Mcl1, in the persistent MPyV infection model. Functional analysis suggested that these genes are associated with inflammation, fibrotic remodeling, and metabolic regulation.

**Conclusion:**

This study characterizes the molecular phenotype and regulatory network of a BKPyVN-Mac subpopulation, providing new insights into BKPyVN pathophysiology and identifying candidate biomarkers for clinical diagnosis.

## 1 Introduction

BK polyomavirus nephropathy (BKPyVN) remains a major cause of renal allograft dysfunction and graft loss after kidney transplantation (1, 2). As no specific antiviral therapy is currently available, clinical management largely relies on monitoring BKPyV replication and reducing immunosuppression, although immunosuppression reduction may increase the risk of alloimmune injury (3). Moreover, early BKPyVN can be difficult to distinguish from other post-transplant complications because of nonspecific clinical and pathological manifestations (4). Therefore, a deeper understanding of the BKPyVN immune microenvironment and the identification of robust disease-associated biomarkers remain important clinical needs.

With increasing recognition of immune evasion and microenvironmental remodeling in BKPyVN, accumulating evidence suggests that macrophages, key regulators of innate immunity, contribute substantially to disease development and progression (5). Given their marked plasticity, macrophages can acquire diverse activation states that coordinate antiviral inflammation, cytokine and chemokine production, tissue repair, and immune regulation within the allograft (6–8). However, previous studies have mainly examined bulk macrophage infiltration or general immune functions, leaving macrophage subtype heterogeneity, disease-associated molecular signatures, and subset-specific functional roles in BKPyVN insufficiently characterized. Furthermore, macrophage-state-specific diagnostic biomarkers and the regulatory programs connecting macrophage activation with persistent inflammation, tissue remodeling, and graft injury in BKPyVN remain poorly understood.

Single-cell RNA sequencing (scRNA-seq) enables high-resolution profiling of gene-expression landscapes at the single-cell level, thereby facilitating the identification of distinct cell subpopulations and regulatory programs that may contribute to BKPyVN-associated immune responses. In this study, we integrated scRNA-seq datasets from BKPyVN and STK samples to characterize macrophage subset heterogeneity and disease-associated phenotypic alterations. We further employed hdWGCNA to identify co-expression modules associated with BKPyVN-associated macrophage populations. By integrating external microarray datasets with machine-learning algorithms, we identified disease-associated signature genes and subsequently validated selected candidates experimentally. These molecular signatures may provide candidate biomarkers for BKPyVN diagnosis and offer new perspectives for future immunologically targeted strategies.

## 2 Materials and methods

### 2.1 Human tissues

This study was conducted in accordance with the Declaration of Helsinki and was approved by the Institutional Review Board of the First Affiliated Hospital of Sun Yat-sen University. Written informed consent was obtained from all participants. A total of five STK biopsy samples and nine BKPyVN biopsy samples were collected for scRNA-seq. BKPyVN was diagnosed based on clinical evidence of BK polyomavirus replication and histopathological confirmation, whereas STK was defined as stable renal allograft biopsy tissue without evidence of BKPyVN or acute rejection. All samples were independently reviewed and diagnosed by experienced clinicians and pathologists. Relevant clinical characteristics and patient information are summarized in **Supplementary Table S1**.

### 2.2 Acquisition and processing of array data

Three microarray datasets (GSE47199, GSE72925, and GSE75693) related to BKPyVN were downloaded from the Gene Expression Omnibus (GEO) database (http://www.ncbi.nlm.nih.gov/geo/) (9–11). A total of 119 samples were included across the three datasets, comprising 30 BKPyVN recipients and 89 STK recipients. The merged expression matrix was corrected for batch effects using the ComBat algorithm implemented in the sva package (12). Boxplots were generated using the ggplot2 package to assess data distribution. The effects of normalization and batch correction were evaluated by comparing the median expression levels and distribution ranges of samples before and after correction. Principal component analysis (PCA) was employed to assess global sample-level variation and the effectiveness of batch effect correction.

The first two principal components were extracted, and scatter plots were generated using the ggpubr package, with samples colored according to dataset source and overlaid with 95% confidence ellipses. The degree of batch effect removal was evaluated by comparing the clustering of samples in PCA space before and after correction.

### 2.3 scRNA-seq: doublet removal and initial clustering

Single-cell RNA-seq datasets were processed and analyzed using Seurat (v5.1.0) (13). Cells with fewer than 200 or more than 4, 000 detected genes, or with >30% mitochondrial reads, were excluded. Doublets were identified and removed using DoubletFinder (v2.0.4) (14). After merging the datasets, the gene expression matrices were log-normalized, and 2, 000 highly variable genes were selected for scaling and principal component analysis (PCA). Batch effects between samples were corrected with Harmony (15). Cells were clustered based on the top 20 principal components at a resolution of 1.0. Finally, cell types were annotated within the resulting clusters according to canonical marker signatures.

### 2.4 Pseudotime trajectory

The R package Monocle (v.2.32.0) was employed to analyze the pseudotime trajectory of myeloid cells in single-cell RNA sequencing data (16, 17). The raw count matrix was extracted from the Seurat object and converted into a Monocle2 CellDataSet object using the newCellDataSet function. Because raw count data were used as input, the CellDataSet object was constructed with lowerDetectionLimit = 0.5 and a negative binomial expression model (expressionFamily = negbinomial.size()). Cell size factors and gene dispersion parameters were estimated using the estimateSizeFactors and estimateDispersions functions, respectively. Expressed genes were detected using the detectGenes function with min_expr = 0.1. Genes used for cell ordering were selected from the Monocle2 dispersion table using the criteria mean_expression ≥ 0.1 and dispersion_empirical ≥ dispersion_fit, and were then applied to the ordering filter using setOrderingFilter. Dimensionality reduction was performed using the reduceDimension function with the DDRTree algorithm and max_components = 2. Cells were ordered along the inferred trajectory using the orderCells function. The inferred pseudotime trajectory was visualized using the plot_cell_trajectory function.

### 2.5 Cell-cell communication analysis

To evaluate intercellular crosstalk, the CellChat R package (v2.2.0) was utilized to model and quantify signaling networks (18). The normalized RNA expression matrix from the Seurat object was used as input, and cell-type annotations were used as cell identity labels. CellChat objects were created separately for the STK and BKPyVN groups using createCellChat, and the human ligand–receptor interaction database (CellChatDB.human) was used as the reference database for all analyses. After standard preprocessing steps were performed to identify overexpressed signaling genes and ligand–receptor interactions, cell–cell communication probabilities were inferred using computeCommunProb with default parameters. Interactions involving cell groups with fewer than 10 cells were excluded using filterCommunication(min.cells = 10). Pathway-level communication probabilities were calculated using compute CommunProbPathway, and the overall communication network was summarized using aggregateNet. For comparative analysis, the STK and BKPyVN CellChat objects were merged using mergeCellChat. Differences in interaction number, interaction strength, incoming and outgoing signaling roles, and pathway activity between the two groups were visualized using compareInteractions, netVisual_heatmap, netAnalysis_signalingRole_scatter, and netAnalysis_signalingRole_heatmap.

### 2.6 High-dimensional WGCNA analysis

hdWGCNA was performed using the hdWGCNA package (v0.4.07) to identify co-expression modules associated with myeloid cell states (19). Genes expressed in at least 5% of cells were retained using SetupForWGCNA with gene_select = “fraction” and fraction = 0.05. To reduce sparsity, metacells were constructed within each cell type and sample (group.by = c(“cell_type”, “orig.ident”)) based on the Harmony reduction, with k = 25 and max_shared = 10. Myeloid cells were then selected for co-expression network construction using SetDatExpr. Candidate soft-thresholding powers were evaluated under an unsigned network setting, and a soft-thresholding power of β = 3 was selected based on the scale-free topology fit and network connectivity. Module eigengenes were calculated using the ModuleEigengenes function. Hub genes were ranked according to eigengene-based connectivity (kME) using ModuleConnectivity, and genes assigned to the grey module were excluded from downstream interpretation.

### 2.7 Predictive model development using machine learning

To construct a robust diagnostic model, the integrated microarray cohort was randomly divided into a development set (70%) and an independent test set (30%) using a fixed random seed (set.seed (123)). Nine machine learning algorithms, including Support Vector Machine (SVM), Partial Least Squares (PLS), Decision Tree Stump (DTS), Logistic Regression, K-Nearest Neighbors (KNN), Gradient Boosting Machine (GBM), Artificial Neural Network (NeuralNet), Generalized Linear Model Boosting (glmBoost), and Extreme Gradient Boosting (XGBoost), were implemented using the R package caret (20–23). Within the development set, we performed repeated 5-fold cross-validation with 10 repeats, yielding 50 resampling iterations for each algorithm. The independent test set was withheld from model training, hyperparameter tuning, and cross-validation, and was used only for final performance evaluation. Cross-validated AUC, sensitivity, and specificity were summarized for internal performance comparison, whereas final model discrimination was assessed on the independent test set using AUC curves. For model interpretation, the DALEX framework was applied to the final selected model to identify predictive genes contributing to model performance. In addition, repeated permutation-based contribution analysis was performed for the top ten SVM-derived feature genes ranked by feature importance scores. Each gene was individually permuted 200 times in the independent test set, and the increase in root mean squared error (ΔRMSE = RMSE_permuted − RMSE_baseline) was used to quantify its contribution to model prediction.

### 2.8 Analysis of immune cell infiltration and pathway activity

The CIBERSORT algorithm was employed to predict immune cell infiltration and calculate the relative percentages of each immune cell type in each sample (24). A bar plot was subsequently generated using the ggpubr package. Box plots illustrating immune cell types were created with the ggplot2 package to compare differences in immune cell distribution between the BKPyVN and STK groups. In addition, single-sample Gene Set Enrichment Analysis (ssGSEA) scores were estimated using the gsva function in the GSVA package (v1.52.3) to evaluate pathway activity across all samples. Differentially activated pathways were identified by comparing ssGSEA scores between groups with Student’s t-test, followed by Benjamini–Hochberg correction. The ssGSEA scores of significantly altered pathways were visualized as box plots using the ggpubr package. Spearman correlation analysis was performed to assess associations between selected diagnostic feature genes and pathway activity scores.

### 2.9 Virtual gene knockout analysis of identified targets

To investigate the potential regulatory impact of identified target genes, virtual gene knockout analysis was performed using the scTenifoldKnk R package (v1.0.3) (25). The raw count matrix extracted from the Seurat object was used as input. To reduce computational complexity while retaining informative genes, highly variable genes were identified using FindVariableFeatures with selection.method = “vst” and nfeatures = 5000. ATF4 and MCL1 were manually added to the highly variable gene set when not already included, and the resulting gene set was used to construct the input matrix. Virtual knockout analyses were performed separately for ATF4 and MCL1 using the scTenifoldKnk function with a fixed random seed (set.seed (123)). Differentially regulated genes resulting from each virtual knockout were extracted from the diffRegulation output, and genes with an FDR-adjusted P value < 0.05 were prioritized for downstream functional enrichment analysis.

### 2.10 MPyV infection model

The MPyV LID strain (ATCC VR-252) was propagated in NIH 3T3 cells according to standard laboratory procedures, and viral titers were determined using qPCR (26). To establish persistent infection, 2-week-old male C57BL/6 mice were inoculated intraperitoneally (i.p.) with 100 μL of MPyV suspension (4 × 10^6^ copies/mL), whereas control mice received an equal volume of sterile saline. Blood samples and kidney tissues were collected at 3, 10, 24, 35, 60, and 80 days post-infection (dpi) for subsequent analysis (n = 3 mice per time point).

### 2.11 Assessment of viral burden

To determine MPyV burden, TaqMan-based quantitative PCR (qPCR) targeting the VP1 gene was performed. The primers and probe used for VP1 quantification are listed in **Supplementary Table S2**. All reactions were performed on an Applied Biosystems 7500 Real-Time PCR System. A serially diluted plasmid containing the full-length MPyV genome was used to generate a standard curve for absolute quantification. Samples without detectable amplification or with Ct values above the detection threshold were considered negative. Viral loads were log10-transformed, normalized to tissue weight or blood volume, and expressed as log10 copies/mg for kidney tissues and log10 copies/mL for blood samples.

### 2.12 Western blot analysis

Kidney tissue samples were homogenized and sonicated in RIPA lysis buffer, and the resulting lysates were centrifuged to collect total protein extracts. Protein concentrations were determined using a BCA assay kit according to the manufacturer’s instructions. Equal amounts of protein were separated by SDS-PAGE and transferred onto PVDF membranes. After blocking with 5% skim milk, the membranes were incubated overnight at 4 °C with primary antibodies against ATF4 (Proteintech, 60035-1-Ig, 1:1000), MCL1 (Proteintech, 16225-1-AP, 1:1000), and GAPDH (Abcam, ab9485, 1:2500). GAPDH served as the internal loading control. After three washes with TBST, the membranes were incubated with horseradish peroxidase (HRP)-conjugated secondary antibodies for 2 h at room temperature. Immunoreactive bands were visualized using an enhanced chemiluminescence (ECL) kit.

### 2.13 RNA extraction and quantitative real-time PCR

Total RNA was extracted from kidney tissues using TRIzol reagent (Invitrogen). RNA concentration and purity were measured using a NanoDrop™ spectrophotometer (Thermo Fisher Scientific), and only samples with an A260/A280 ratio of 1.8–2.1 were used for subsequent analyses. One microgram of total RNA was reverse-transcribed into cDNA using a cDNA synthesis kit (TaKaRa). mRNA expression levels were quantified by quantitative real-time PCR (qPCR) using cDNA as the template on an Applied Biosystems™ 7500 Real-Time PCR System (Thermo Fisher Scientific). The qPCR primer sequences are provided in **Supplementary Table S2**. Reactions were prepared using SYBR Green Master Mix (Bimake) and performed according to the manufacturer’s recommended cycling conditions. Target gene expression was normalized to the corresponding endogenous control gene, Gapdh for mouse samples and GAPDH for human samples. Relative expression levels were calculated using the 2^−ΔΔCt method. For the mouse MPyV time-course experiment, the corresponding time-matched mock-infected group was used as the calibrator at each selected time point. For human kidney biopsy validation, the STK was used as the reference group.

### 2.14 Immunohistochemical staining

Formalin-fixed, paraffin-embedded human renal allograft biopsy specimens were sectioned at a thickness of 4 μm. After deparaffinization and rehydration, heat-induced antigen retrieval was performed in citrate buffer (pH 6.0). The sections were blocked for 30 min and incubated overnight at 4 °C with primary antibodies against ATF4 (Proteintech, 60035-1-Ig, 1:100) or MCL1 (Proteintech, 16225-1-AP, 1:100). After incubation with the corresponding secondary antibodies, immunoreactivity was visualized using 3, 3′-diaminobenzidine, followed by hematoxylin counterstaining. Representative images were acquired under identical imaging conditions.

### 2.15 Statistical analysis

Data processing and visualization were performed using R software (version 4.4.1) and GraphPad Prism (version 9.0). Continuous experimental data are presented as the mean ± SD or mean ± SEM, as indicated in the figure legends. For comparisons between two groups, unpaired Student’s t-test was used unless otherwise specified. For RT-qPCR analysis, statistical comparisons were performed using ΔCt values. Mouse MPyV time-course qPCR data were analyzed by ordinary two-way ANOVA followed by Bonferroni-adjusted comparisons between infected and time-matched mock-infected groups at each selected time point. Correlation analyses were performed using Spearman correlation analysis. For high-dimensional analyses, including differential expression, enrichment, ssGSEA, gene–pathway correlation, and in silico knockout analyses, P values were adjusted using the Benjamini–Hochberg method to control the false discovery rate. Statistical significance in these analyses was defined as an FDR-adjusted P value < 0.05.

## 3 Results

### 3.1 Single-cell RNA sequencing characterizes the cellular atlas of BKPyVN

To characterize changes in cellular composition associated with BKPyVN, we analyzed single-cell RNA sequencing data from STK and BKPyVN tissues. After quality control, 44, 459 high-quality cells were retained for downstream analyses. Using the Seurat pipeline, 14 major cell populations were identified, annotated based on canonical marker genes, and visualized using t-SNE (**Figures 1A–C**). These annotated cell types included proximal tubular cells (PTC), loop of Henle cells (LOH), collecting duct cells (CD), B cells (B), natural killer cells (NK), CD4+ T cells (CD4+ T), CD8+ T cells (CD8+ T), myeloid cells (MD), mast cells (MT), neutrophils (NE), endothelial cells (EC), fibroblasts (FB), plasma cells (PC), and cycling cells (CC). Representative marker genes for each cell type are shown in **Figure 1E**. Notably, myeloid cells showed a marked difference in relative abundance between STK and BKPyVN tissues (**Figure 1D**).

**Figure 1:**
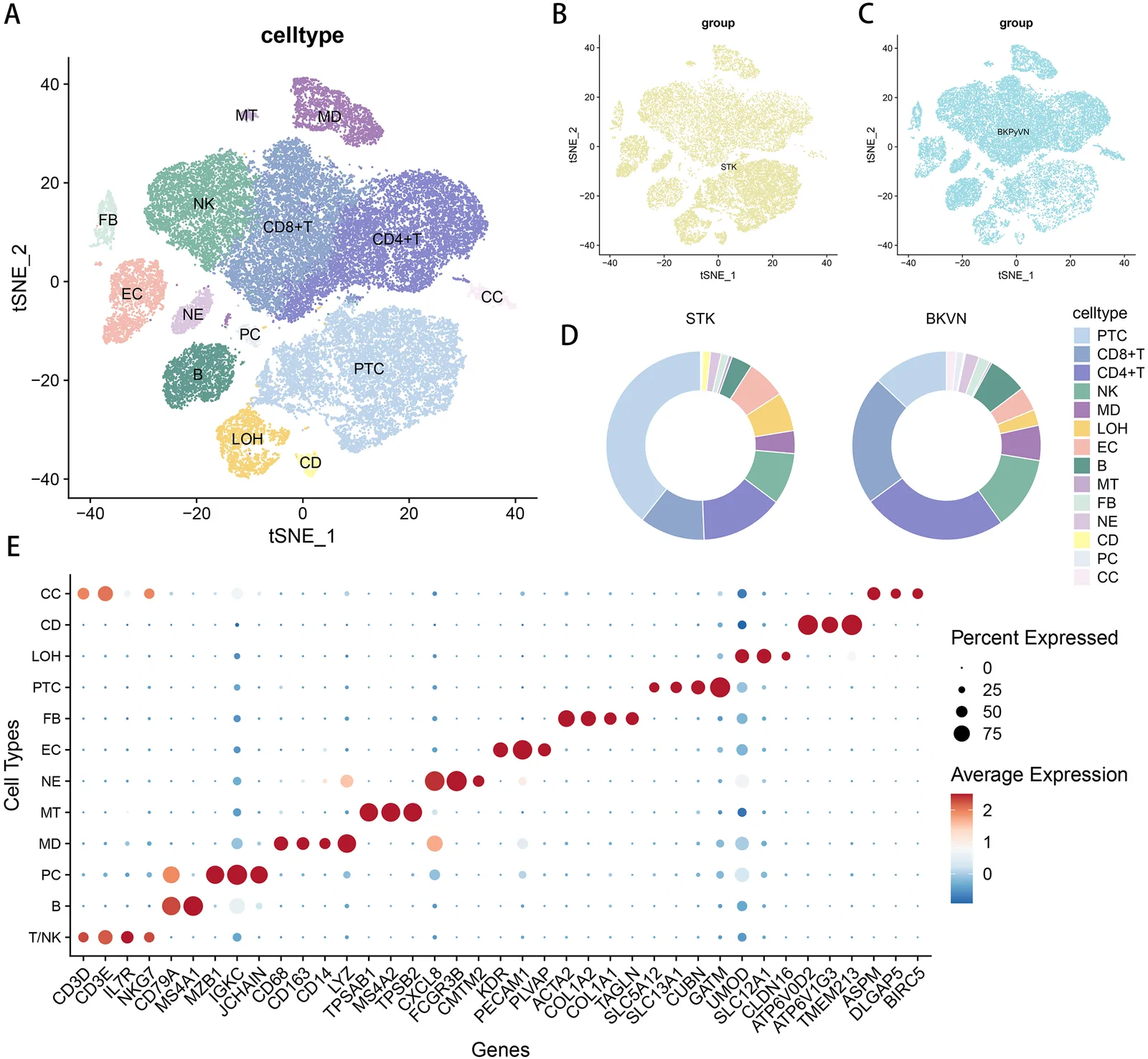
Identification of cell types and alterations in cellular proportions in BKPyVN and STK tissues. **(A)** t-SNE clustering analysis of single cells from 5 STK and 9 BKPyVN tissue samples identified 14 major cell clusters. **(B, C)** t-SNE plots illustrating the group origin of cells, with colors representing the corresponding groups. **(D)** Relative cellular composition of STK and BKPyVN tissues. **(E)** Dot plot displaying the expression of canonical marker genes across distinct cell populations. Dot size represents the percentage of cells expressing each gene in each cluster, whereas color intensity indicates the average expression level.

### 3.2 Single-cell RNA sequencing analysis reveals the heterogeneity of myeloid cells in BKPyVN

Focusing on myeloid cells, dimensionality reduction and secondary clustering were performed, leading to the identification of 12 distinct cell subsets based on classical markers (**Figures 2A–D**). These subsets included six macrophage subtypes (Macro1–Macro6), four dendritic cell subtypes (pDC, cDC1–cDC3), and two monocyte subtypes (Mono1–Mono2). Compared with the STK group, Macro1, Macro3, and Macro4 were increased in BKPyVN (**Figure 2B**). Cellular preference analysis further supported this pattern (**Figure 2C**). Consequently, Macro1, Macro3, and Macro4 were defined as BKPyVN-associated macrophages (BKPyVN-Mac), while the remaining macrophages were categorized as Other-Mac. To characterize BKPyVN-Mac, we identified their top marker genes (**Supplementary Table S3**). Representative markers, including OLR1, TAP1, CXCL9, GBP5, STAT1, FN1, IL1RN, LGALS3, and CLEC4E, suggest that BKPyVN-Mac represents a disease-associated macrophage state involving interferon-related antiviral responses, antigen processing and presentation, macrophage activation, adhesion, and tissue remodeling. Crucially, the expansion of the BKPyVN-Mac population was positively correlated with clinical disease progression, showing a continuous increase from STK to AST Stage B1 and peaking in AST Stage B2 (**Figure 2E**). Enrichment analysis revealed that BKPyVN-Mac exhibited significant upregulation in several GO pathways, including cytoplasmic translation, lymphocyte activation, leukocyte activation, cell killing, cell–substrate junction, and cadherin binding (**Figure 2F**).

**Figure 2:**
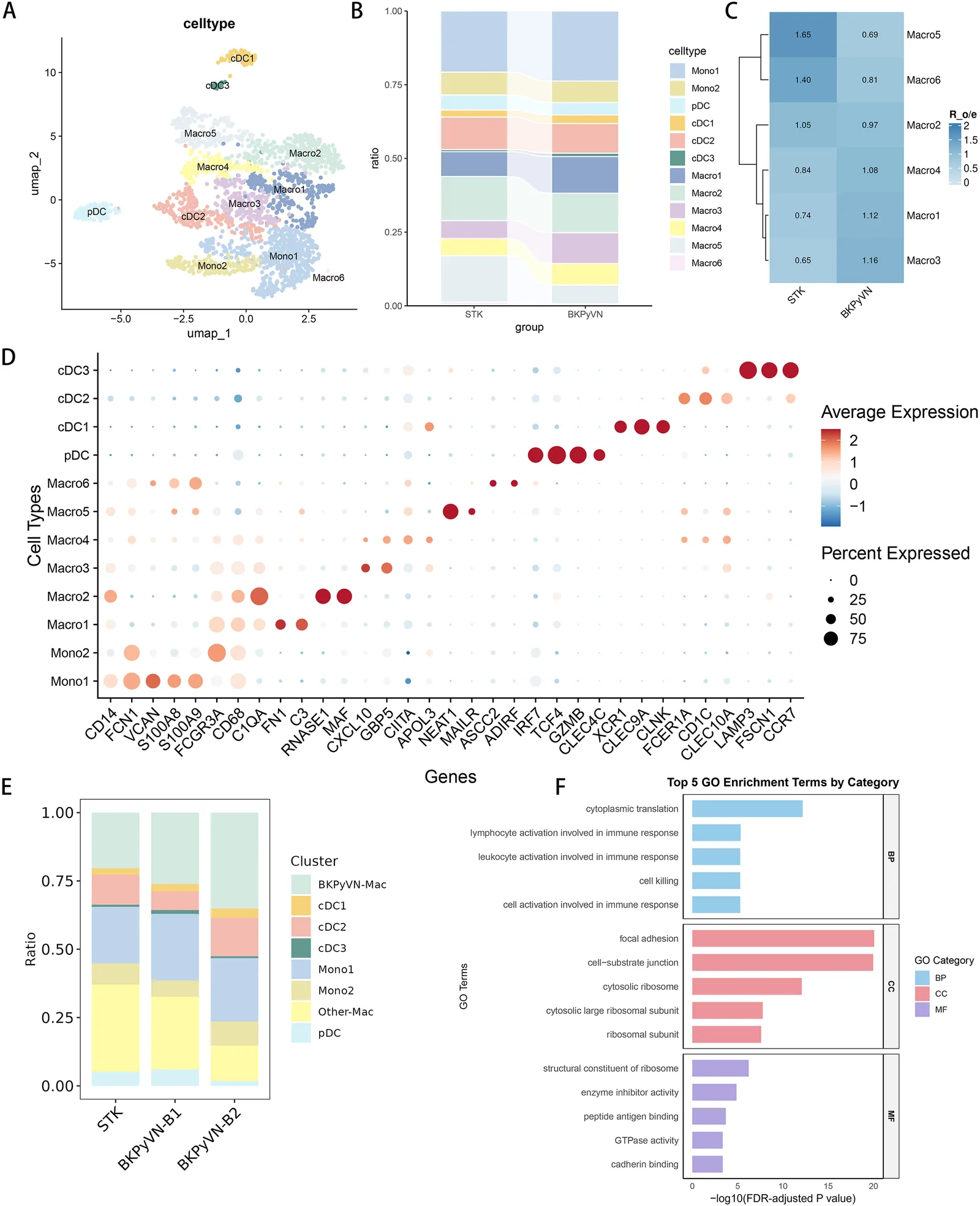
Single-cell analysis reveals the heterogeneity of myeloid cells in BKPyVN. **(A)** UMAP plot illustrating the distribution of 12 myeloid cell populations in STK and BKPyVN tissues. **(B)** Cell proportion plot showing compositional differences in the 12 myeloid cell populations between STK and BKPyVN. **(C)** Cell-preference heatmap showing group-specific enrichment patterns of distinct myeloid subsets. The color scale and values represent the ratio of observed to expected cell numbers (Ro/e). **(D)** Dot plot displaying the expression of marker genes across the 12 myeloid cell types. Dot size indicates the percentage of cells expressing each gene, whereas color intensity indicates the scaled average expression level. **(E)** Association between myeloid cell populations and disease stage. **(F)** GO enrichment analysis of BKPyVN-Mac marker genes. The top enriched terms in the biological process (BP), cellular component (CC), and molecular function (MF) categories are displayed. The length of each bar represents the −log10-transformed FDR-adjusted P value.

### 3.3 BKPyVN-Mac: pseudotime trajectory and cellular communication

We next integrated pseudotime and CellChat analyses to examine myeloid-cell state transitions and intercellular communication networks in BKPyVN. Pseudotime analysis revealed the unique position of BKPyVN-Mac along the developmental trajectory (**Figures 3A, B**). The formation of BKPyVN-Mac was observed at the beginning of the trajectory as well as at branches 2 and 1, indicating a specific developmental process (**Figures 3C, D**). CellChat analysis showed that, compared with STK tissues, BKPyVN tissues exhibited a higher inferred number of cell–cell interactions and greater overall interaction strength (**Figure 4A**). Moreover, BKPyVN-Mac showed stronger inferred interactions with multiple cell populations, with increased incoming and outgoing signaling strengths (**Figures 4B–D**). Compared with Other-Mac, BKPyVN-Mac exhibited higher inferred signaling activity in the ADGRE, SPP1, FN1, ANNEXIN, SELL, and ANGPTL pathways (**Figure 4E**). Similar increases were observed in both incoming and outgoing signaling patterns of BKPyVN-Mac for these pathways (**Supplementary Figures S1A, B**).

**Figure 3:**
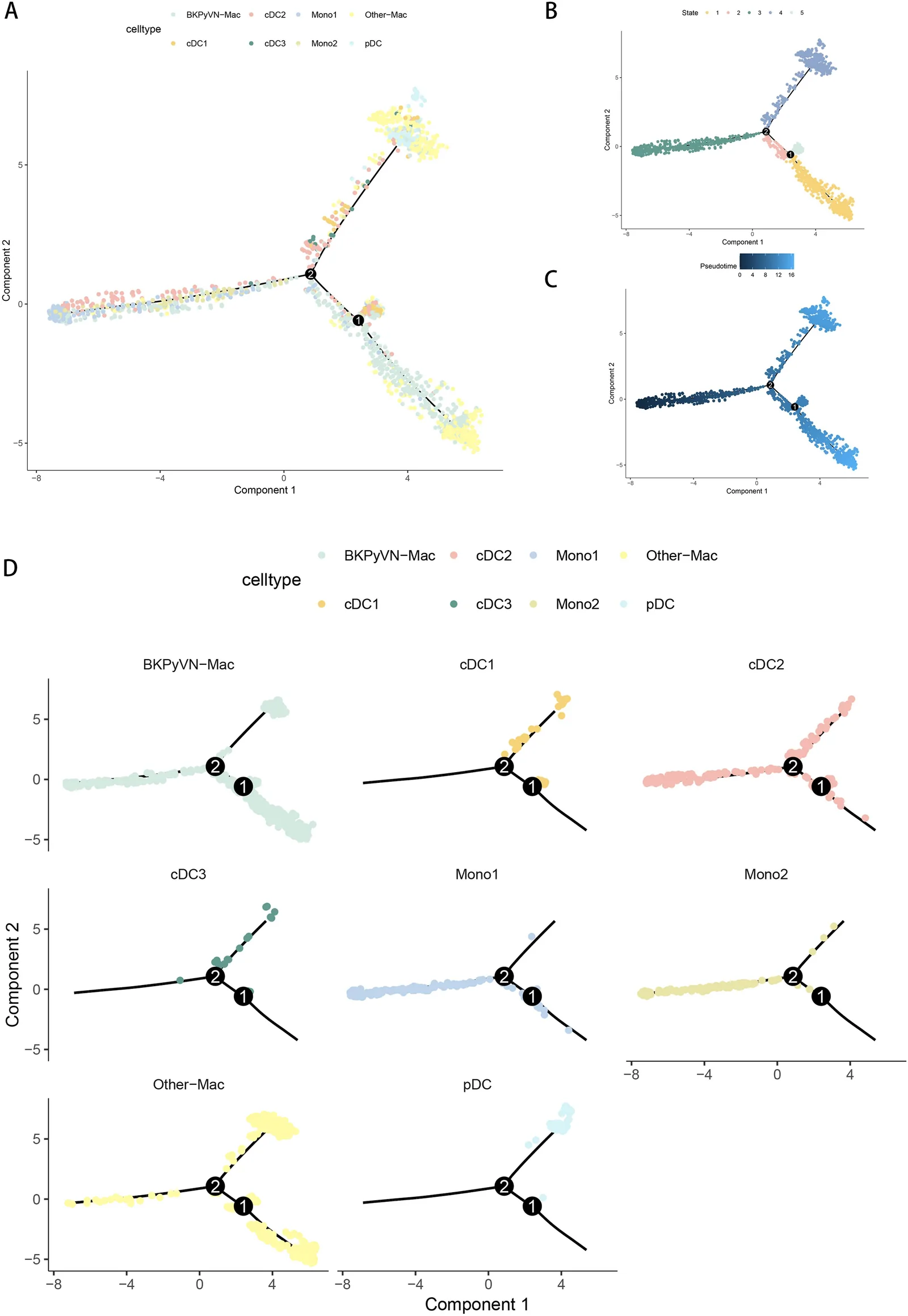
Single-cell pseudotime trajectory analysis reveals the state-transition dynamics of BKPyVN-Mac. **(A)** Visualization of the myeloid cell state-transition trajectory inferred by Monocle2, colored by specific cell type. **(B)** Distribution of cells across distinct trajectory states along the inferred pseudotime path. **(C)** Projection of pseudotime values indicating pseudotemporal progression from early to late states. **(D)** Individual trajectories displayed separately for each cell type to illustrate cell-type-specific distributions along the inferred trajectory.

**Figure 4:**
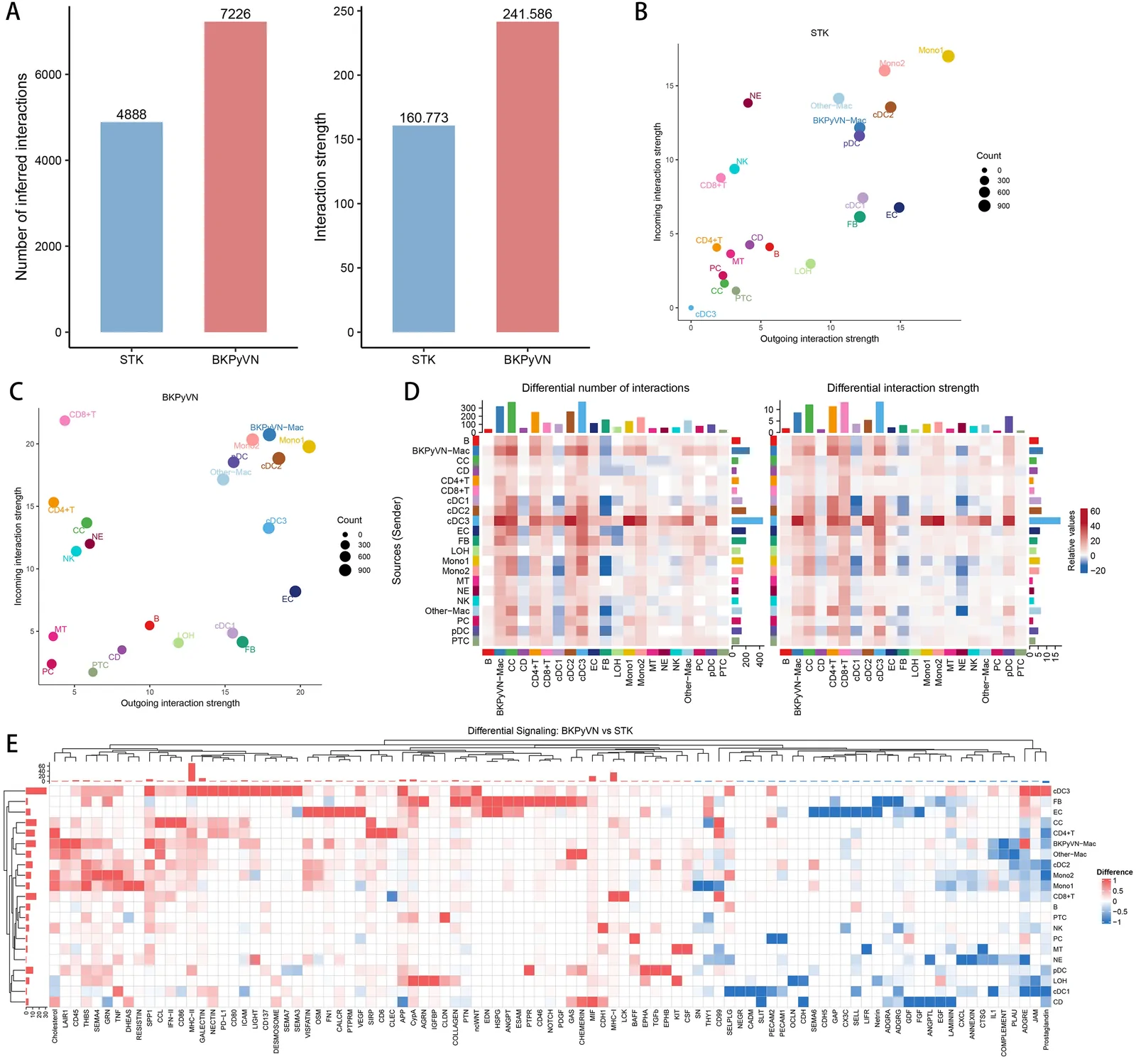
CellChat analysis of BKPyVN-Mac. **(A)** Bar plots showing differences in the overall number and strength of inferred cell–cell interactions between the STK and BKPyVN groups. **(B, C)** Two-dimensional signaling role plots showing the incoming and outgoing interaction strengths of different cell populations in the STK and BKPyVN groups. **(D)** Heatmap showing the inferred interaction intensity among different cell types. Marginal bar plots indicate the relative contribution of each cell population to interaction number or strength. The color scale indicates relative interaction strength. **(E)** Heatmap showing differential inferred signaling pathway activity across cell types between the STK and BKPyVN groups.

Together, these findings suggest that BKPyVN-Mac is not simply an expanded macrophage population, but a disease-associated macrophage state potentially shaped by persistent polyomavirus-associated immune stimulation. Enhanced ADGRE/SELL and SPP1/FN1 signaling may further link macrophage adhesion and recruitment to inflammatory crosstalk and tissue remodeling in BKPyVN.

### 3.4 HdWGCNA reveals module hub genes in BKPyVN-Mac

We employed high-dimensional weighted gene co-expression network analysis (hdWGCNA) to explore the molecular programs associated with BKPyVN-Mac. Based on the scale-free topology model fit and network connectivity evaluation, β = 3 was selected as the lowest soft-thresholding power that reached the predefined fit threshold while maintaining relatively preserved network connectivity (**Figure 5A**). Using this parameter, we constructed 15 co-expression modules in MD cells (**Figure 5B**). Among the 15 modules, MD2, MD4, MD9, MD13, and MD14 showed the strongest association with BKPyVN-Mac (**Figure 5C**). Additionally, MD8, MD2, MD6, MD10, MD12, and MD11 formed one highly correlated module cluster, whereas MD4, MD13, MD9, and MD14 formed another correlated module cluster (**Figure 5D**). Subsequently, the top ten genes in each module, ranked by eigengene-based connectivity (kME), were visualized to identify highly connected module hub genes, defined here as genes with strong correlation to the corresponding module eigengene (**Figure 5E**). Hub-gene networks were then constructed for modules MD2, MD4, MD9, MD13, and MD14 (**Supplementary Figures S2A-E**).

**Figure 5:**
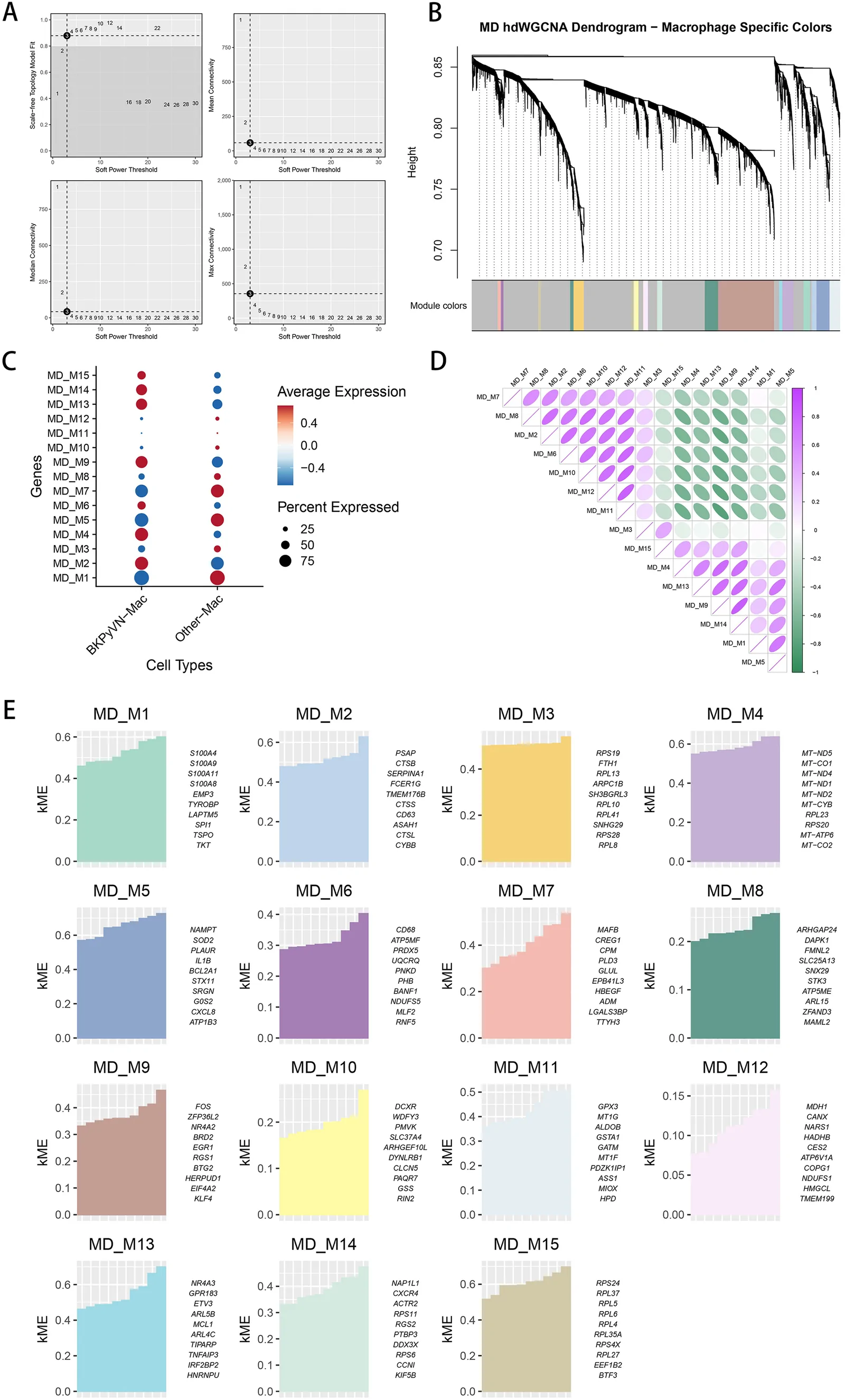
Construction of Macrophage co-expression networks using hdWGCNA and identification of BKPyVN-Mac specific modules. **(A)** Evaluation of soft-thresholding powers for hdWGCNA network construction. The scale-free topology model fit index and network connectivity metrics, including mean, median, and maximum connectivity, were assessed across candidate powers. Black dots indicate the selected power β = 3 and its corresponding values. β = 3 was selected as the lowest power reaching the scale-free topology fit criterion (fit index > 0.8) while retaining greater network connectivity than larger powers. **(B)** hdWGCNA dendrogram showing 15 distinct modules. **(C)** Bubble plot illustrating module scores across the 15 modules in BKPyVN-Mac and Other-Mac cells. **(D)** Correlation heatmap showing relationships among gene modules. **(E)** Hub genes with high intramodular connectivity are displayed for each module.

### 3.5 Identification of diagnostic signature genes for BKPyVN through integrative transcriptomic analysis and machine learning

Comparison of expression distributions before and after batch correction showed that batch effects across the integrated microarray datasets were effectively reduced (**Figures 6A, B**). Differential expression analysis was then performed on genes derived from the BKPyVN-Mac-associated modules, including MD2, MD4, MD9, MD13, and MD14 (**Figure 6C**). GO enrichment analysis of the differentially expressed module genes revealed significant enrichment in functional terms related to protein synthesis, RNA binding and processing, regulation of apoptosis, cell adhesion, and cell junction organization (**Figure 6D**). Candidate genes with an individual AUC greater than 0.80 are shown in **Supplementary Figures S3A–M**.

**Figure 6:**
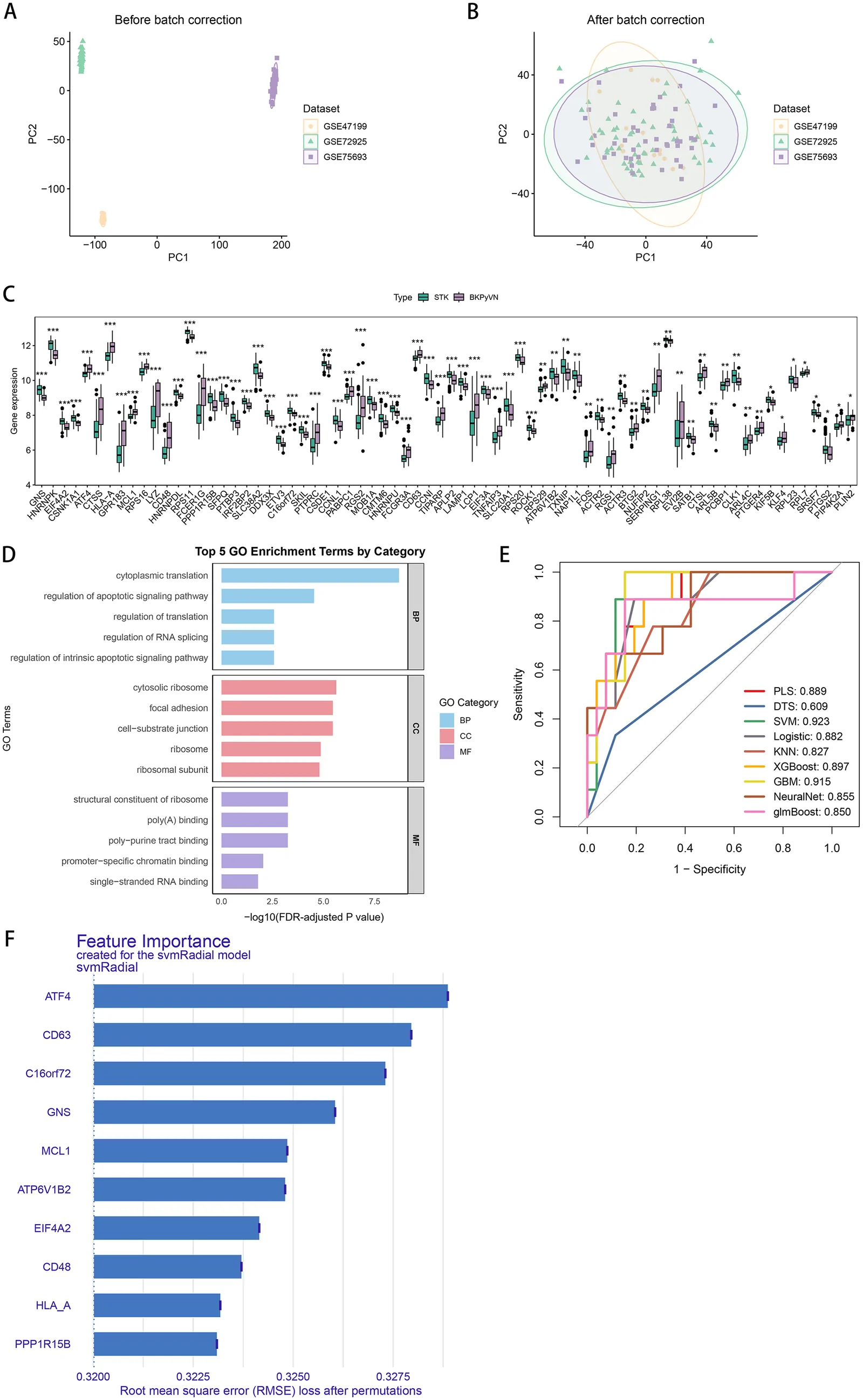
Screening of diagnostic biomarkers for BKPyVN by integrating single-cell and microarray data with machine learning. **(A, B)** Comparison of data distribution before and after batch effect correction, demonstrating the effectiveness of data integration and normalization. **(C)** Box plots showing the expression of FDR-significant genes from the key BKPyVN-Mac-associated co-expression modules, including MD2, MD4, MD9, MD13, and MD14. Gene expression differences between groups were assessed using the Wilcoxon rank-sum test, followed by Benjamini–Hochberg FDR correction. Significance labels indicate FDR-adjusted P values: *FDR < 0.05, **FDR < 0.01, and ***FDR < 0.001. **(D)** GO enrichment analysis of differentially expressed genes from the key BKPyVN-Mac-associated co-expression modules. The top five enriched terms in each GO category are shown, including BP, CC, and MF. Bar length indicates the −log10-transformed FDR-adjusted P value. **(E)** Performance comparison of nine machine learning algorithms, with the SVM model achieving the highest AUC. **(F)** The top 10 feature genes identified by the best-performing SVM model, ranked according to their importance scores.

Subsequently, nine machine-learning algorithms were compared to identify diagnostic feature genes for BKPyVN(**Figure 6E**). Among these algorithms, SVM achieved the highest AUC in the independent test set. Repeated cross-validation within the development set further showed that SVM maintained stable performance, with a mean cross-validated AUC of 0.9089 ± 0.0921, which was comparable to that of PLS, the algorithm with the highest mean cross-validated AUC (0.9127 ± 0.0782). The difference between the independent test AUC and the mean cross-validated AUC for SVM was small (0.0142), supporting the consistency between internal resampling and independent test-set performance. The detailed stability comparison of all nine algorithms is provided in **Supplementary Table S4**.

Because SVM achieved the highest independent test-set AUC while maintaining stable cross-validated performance, it was retained as the final model for feature importance analysis. The top-ranked feature genes identified by the SVM model were ATF4, CD63, C16orf72, GNS, MCL1, ATP6V1B2, EIF4A2, CD48, HLA-A, and PPP1R15B (**Figure 6F**). The chromosomal locations of the top five genes ranked by importance were subsequently visualized (**Supplementary Figure S4A**). The top 10 feature genes identified by the other machine-learning algorithms are shown in **Supplementary Figures S4B–I**. To further quantify the model contribution of these SVM-derived feature genes, we performed repeated permutation-based contribution analysis on the ten genes shown in **Figure 6F**. The detailed contribution weights are summarized in **Supplementary Table S5**.

### 3.6 Distinct immune cell infiltration landscape and exploratory gene–immune cell associations in BKPyVN

CIBERSORT was applied to the integrated expression matrix from three GEO datasets to estimate relative immune-cell fractions. BKPyVN samples exhibited significantly higher estimated fractions of CD8+ T cells, activated memory CD4+ T cells, and M1 macrophages than STK samples (**Figures 7A, B**). Pairwise correlation analysis further showed that the estimated M1 macrophage fraction was positively associated with monocytes and negatively associated with naive CD4+ T cells and naive B cells, whereas the estimated M2 macrophage fraction was negatively associated with T follicular helper cells and regulatory T cells (**Supplementary Figure S4J**).

**Figure 7:**
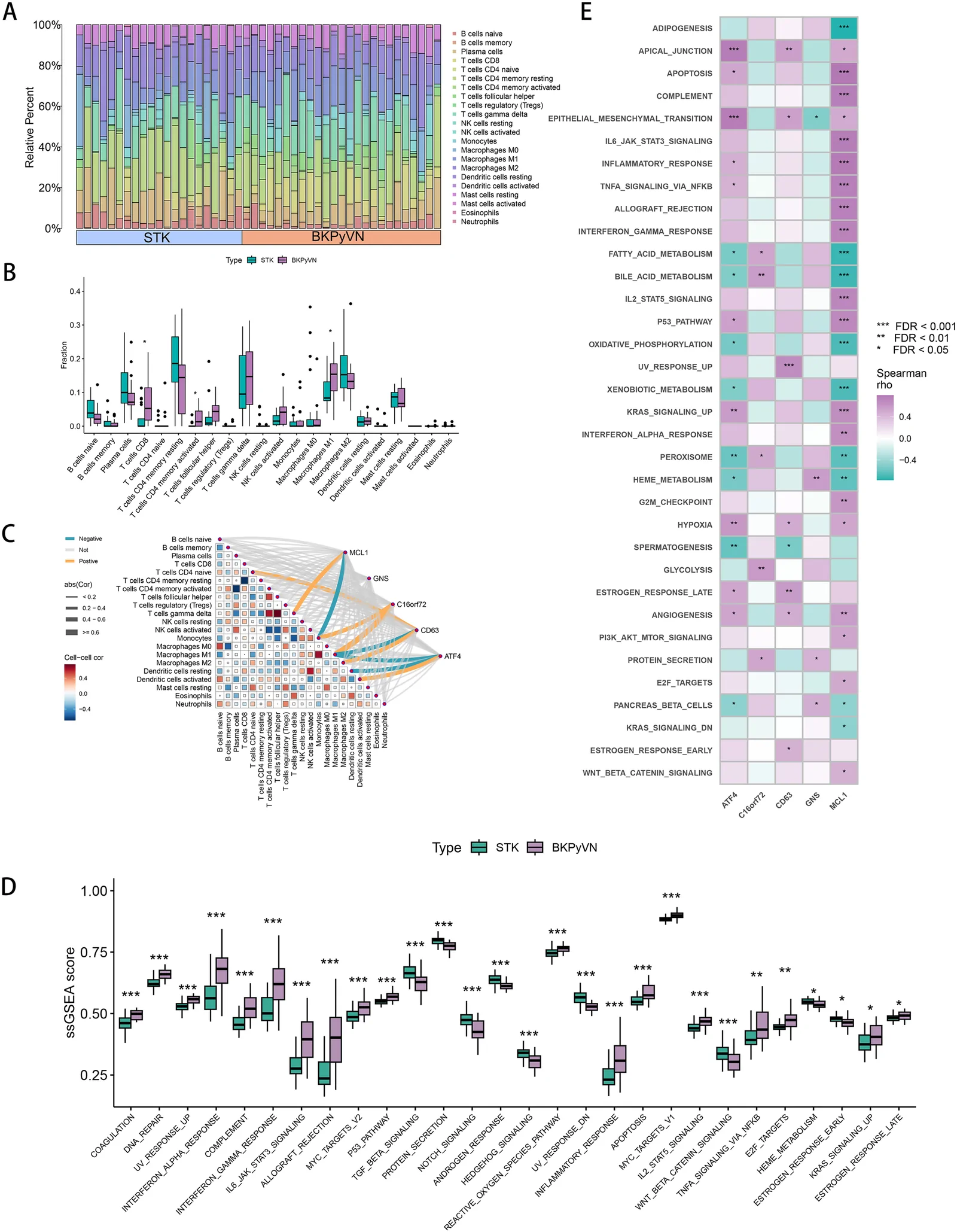
Immune infiltration landscape and signaling pathway activation characteristics in BKPyVN correlated with signature genes. **(A)** Stacked bar plot showing the relative fractions of 22 immune cell types estimated by the CIBERSORT algorithm in individual STK and BKPyVN samples. **(B)** Box plots comparing the relative fractions of 22 immune cell types between STK and BKPyVN samples. **(C)** Spearman correlation analysis between the expression levels of key diagnostic signature genes (ATF4, C16orf72, CD63, MCL1, GNS) and the infiltration abundance of immune cells. **(D)** Box plots showing ssGSEA scores of FDR-significant pathways between STK and BKPyVN samples. Only pathways with FDR < 0.05 are displayed. **(E)** Heatmap showing Spearman correlations between the expression levels of the top five SVM-derived diagnostic feature genes and ssGSEA pathway scores. The color scale represents Spearman’s rank correlation coefficient. *, **, and *** indicate FDR < 0.05, FDR < 0.01, and FDR < 0.001 after Benjamini–Hochberg correction, respectively.

We next explored the relationships between key diagnostic feature genes and CIBERSORT-derived immune-cell fractions. Several gene–immune cell pairs showed nominal associations based on unadjusted P values, including ATF4 with dendritic cell subsets and M1 macrophages, C16orf72 with M1 macrophages and monocytes, CD63 with macrophage subsets and naive CD4+ T cells, and MCL1 with monocytes and γδ T cells (**Figure 7C**; **Supplementary Figure S5**). However, these associations did not remain statistically significant after Benjamini–Hochberg false discovery rate correction and were therefore interpreted as exploratory trends rather than statistically significant findings.

### 3.7 Identification of BKPyVN pathway signatures using ssGSEA and functional annotation of key genes

ssGSEA analysis revealed widespread differences in pathway activity between BKPyVN and STK tissues (**Figure 7D**). The differentially active pathways were mainly enriched in immune-inflammatory and antiviral programs, including interferon responses, IL6–JAK–STAT3 signaling, TNFA signaling via NF-κB, complement activation, inflammatory response, and allograft rejection. Pathways related to cellular stress and injury responses, such as apoptosis, the p53 pathway, reactive oxygen species pathway, and DNA repair, were also altered, indicating coordinated activation of inflammatory, antiviral, and stress-response programs in BKPyVN tissues.

We next examined the associations between the top five SVM-derived diagnostic feature genes and ssGSEA pathway scores using Spearman correlation analysis (**Figure 7E**). Among these genes, ATF4 and MCL1 showed biologically interpretable and complementary pathway-association patterns. ATF4 was mainly associated with cellular stress and tissue injury/remodeling-related pathways, including hypoxia, the p53 pathway, apoptosis, epithelial–mesenchymal transition, and apical junction, and was negatively associated with metabolic programs such as fatty acid metabolism and oxidative phosphorylation. In contrast, MCL1 showed broader positive associations with immune-inflammatory and survival-related pathways, including allograft rejection, inflammatory response, IL6–JAK–STAT3 signaling, TNFA signaling via NF-κB, interferon responses, complement activation, apoptosis, and PI3K–AKT–mTOR signaling.

To further quantify these associations within BKPyVN samples, we performed individual linear regression analyses for ATF4/MCL1-associated pathways identified by FDR-adjusted Spearman correlation analysis (**Supplementary Figure S6**). Together with SVM feature importance and permutation-based contribution analysis, these pathway-level associations supported the prioritization of ATF4 and MCL1 for subsequent experimental validation.

### 3.8 Experimental validation of ATF4/MCL1 expression in polyomavirus infection

To validate the bioinformatics findings, we examined the dynamic expression of Atf4/Mcl1 in a mouse model of persistent MPyV infection and evaluated ATF4/MCL1 expression in human kidney biopsies. First, we established a persistent MPyV infection model in mice. Viral load dynamics in kidney tissues and blood are shown in **Figure 8A**. Blood viral load peaked at 24 dpi and subsequently declined to below the detection limit, whereas kidney viral load peaked at 35 dpi and remained high thereafter, confirming the establishment of persistent renal infection.

**Figure 8:**
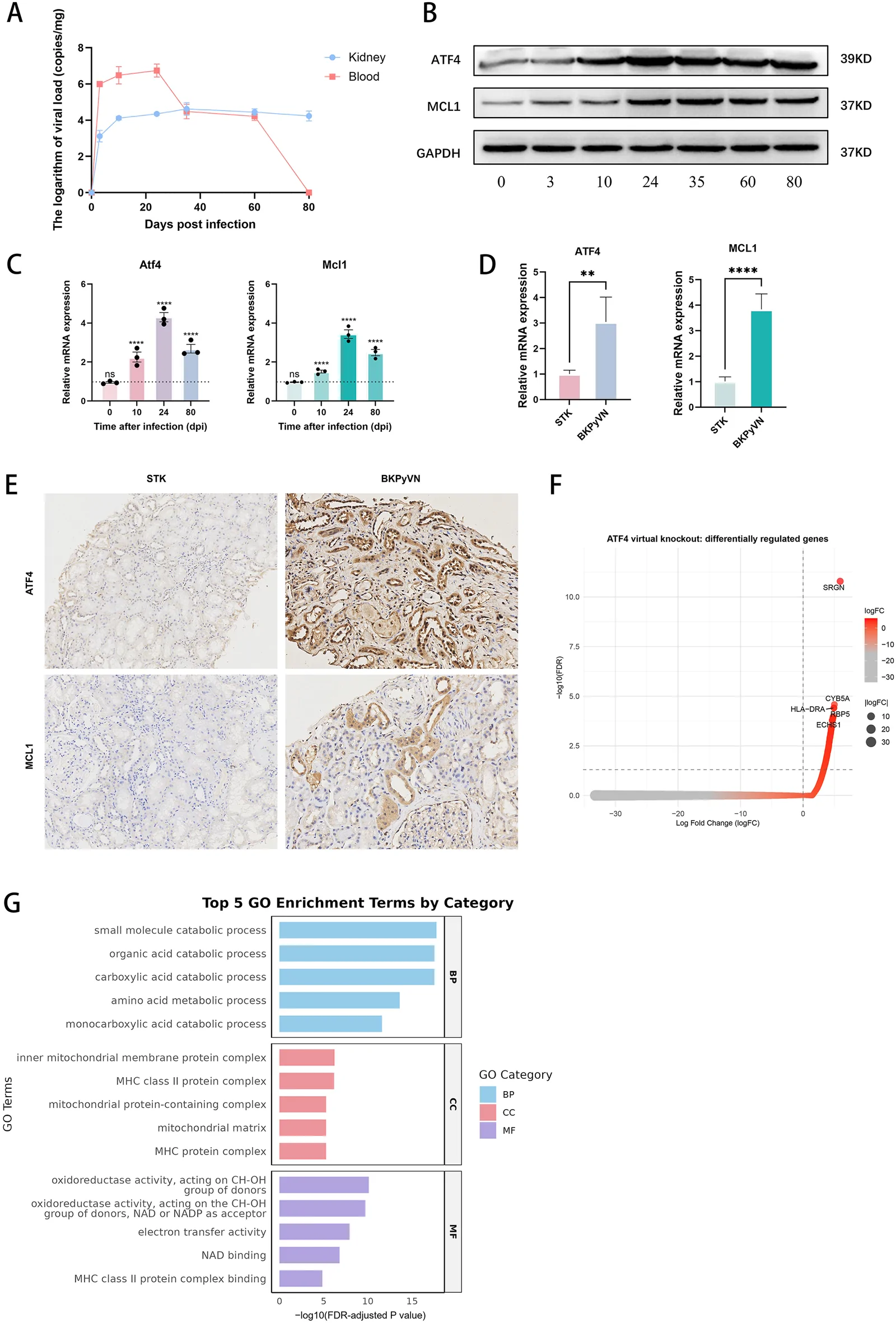
Experimental validation and in silico gene knockout analysis of key biomarkers in BKPyVN. **(A)** Viral load kinetics in blood and kidney tissues following MPyV infection in C57BL/6 mice over 80 days. Data are presented as the mean ± SD, with n = 3 mice per time point. Viral loads are expressed as log10 copies/mg for kidney tissues and log10 copies/mL for blood samples. **(B)** Representative western blot images showing the time-course changes in ATF4 and MCL1 protein expression in kidney tissues at 0, 3, 10, 24, 35, 60, and 80 dpi following MPyV infection. GAPDH was used as the loading control. **(C)** RT-qPCR analysis of relative Atf4 and Mcl1 mRNA expression levels in kidney tissues at representative time points, including 0, 10, 24, and 80 dpi. Gapdh was used as the internal reference gene, and relative expression was calculated using the 2^-ΔΔCt method with the corresponding time-matched mock-infected group as the calibrator. The dashed line at y = 1 indicates the normalized expression level of time-matched mock controls. Data are presented as the mean ± SEM, with each dot representing one mouse (n = 3 mice per group at each selected time point). Significance labels indicate Bonferroni-adjusted P values versus time-matched mock controls. **(D)** Relative mRNA expression levels of ATF4 and MCL1 in human kidney biopsies from STK and BKPyVN patients (STK, n = 5; BKPyVN, n = 6). **(E)** Representative immunohistochemical staining of ATF4 and MCL1 in human renal allograft biopsies from the STK and BKPyVN groups. Scale bar = 100 μm. **(F)** Bubble plot showing differentially regulated genes following ATF4 virtual knockout in BKPyVN scRNA-seq data. Significance was determined using FDR-adjusted P values, with the horizontal dashed line indicating FDR = 0.05. Labeled genes represent the top five FDR-significant genes. Dot size indicates |logFC|, and dot color represents logFC. **(G)** GO enrichment analysis of genes significantly affected by ATF4 virtual knockout. Genes with FDR < 0.05 in the scTenifoldKnk differential regulation analysis were included. The top five enriched terms in each GO category are shown, including BP, CC, and MF. Bar length indicates the −log10-transformed FDR-adjusted P value.

To determine whether ATF4/MCL1 expression was temporally associated with persistent renal polyomavirus infection, we assessed ATF4/MCL1 protein expression across the full experimental time course and measured Atf4/Mcl1 mRNA expression at representative time points. Time-course western blot analysis performed at 0, 3, 10, 24, 35, 60, and 80 dpi showed that ATF4 protein was induced at 24 dpi and remained elevated thereafter, with a modest decline at later time points (**Figure 8B**). MCL1 protein showed a marked increase at 24 dpi and remained elevated throughout the observation period. Consistently, RT-qPCR analysis performed at 0, 10, 24, and 80 dpi showed that Atf4 mRNA expression was significantly increased after infection, reached the strongest relative induction at 24 dpi, and remained elevated at 80 dpi compared with time-matched mock controls (**Figure 8C**). Mcl1 mRNA expression was also upregulated during infection and maintained an elevated level at the late infection stage. For RT-qPCR analysis, the corresponding time-matched mock-infected group was used as the calibrator at each time point. To assess the clinical relevance of these findings, we measured ATF4 and MCL1 mRNA expression in kidney biopsies from patients with BKPyVN and STK. Both transcripts were significantly upregulated in BKPyVN compared with STK (**Figure 8D**). We subsequently performed immunohistochemical staining to evaluate ATF4 and MCL1 protein expression in human renal allograft biopsy specimens. Representative BKPyVN biopsies exhibited stronger and more extensive immunoreactivity for both ATF4 and MCL1 than STK (**Figure 8E**). Together, these transcript-level and histological findings provide complementary evidence supporting the clinical relevance of ATF4/MCL1 upregulation in BKPyVN.

### 3.9 Virtual gene knockout analysis of ATF4 and MCL1

To explore the predicted regulatory networks associated with the identified signature genes within the BKPyVN microenvironment, we performed in silico virtual knockout analysis using the scTenifoldKnk algorithm. Following virtual knockout of ATF4, 179 genes were significantly differentially regulated (FDR-adjusted P value < 0.05, **Supplementary Table S6**). **Figure 8F** shows the top 10 significantly perturbed genes ranked by absolute log fold change (|logFC|). Notably, SRGN showed the largest absolute change among the significantly perturbed genes. Metabolism-related genes such as NAMPT and ECHS1 and antigen-presentation-related genes such as HLA-DRA, CD74, and HLA-DRB1 were among the significantly perturbed genes. GO enrichment analysis of the significantly differentially regulated genes (FDR-adjusted P value < 0.05) revealed significant enrichment in biological processes related to small-molecule, organic-acid, and amino-acid catabolism. Significant enrichment was also observed in cellular component terms related to the inner mitochondrial membrane and MHC class II protein complexes, as well as molecular function terms related to oxidoreductase activity and NAD binding (**Figure 8G**).

In contrast, virtual knockout of MCL1 produced a more restricted perturbation profile, with only seven genes showing significant differential regulation (FDR-adjusted P value < 0.05, **Supplementary Table S6**). Among these genes, the dual-specificity phosphatases DUSP2 and DUSP4 showed marked perturbation. MCL1 perturbation was also associated with significant changes in CXCR4, a chemokine receptor, and the transcription factors NR4A2 and CREM.

## 4 Discussion

Post-transplant BKPyVN is characterized by chronic inflammation driven by persistent viral infection and dysregulated host immune responses (27). This interaction may create a self-perpetuating cycle of viral replication and inflammation, ultimately contributing to progressive allograft injury (28, 29). Macrophages play a critical role in shaping the immune microenvironment of renal allografts; however, their subset heterogeneity and specific contribution to BKPyVN pathogenesis remain insufficiently characterized (5). To address this gap, we analyzed scRNA-seq data from BKPyVN allograft biopsies and identified a disease-associated macrophage population, termed BKPyVN-Mac. We further characterized its molecular signature, cellular communication patterns, and predicted pathogenic relevance within the BKPyVN microenvironment.

Because studies specifically characterizing macrophage subpopulations in BKPyVN remain limited, we mainly compared the BKPyVN-Mac marker profile with macrophage states previously reported in renal injury, renal fibrosis, and allograft inflammation. BKPyVN-Mac shared several features related to activation, adhesion, and tissue remodeling with inflammatory, injury-associated, and profibrotic macrophage programs reported in kidney injury and fibrosis, as reflected by the expression of genes such as OLR1, CD44, FN1, AREG, LGALS3, IL1RN, and CLEC4E (30–33). By contrast, BKPyVN-Mac showed a prominent antiviral and antigen-processing signature, including TAP1, GBP1, GBP2, GBP5, STAT1, and CXCL9. This pattern was more consistent with an interferon-activated macrophage state potentially shaped by persistent polyomavirus infection within the renal allograft microenvironment. Therefore, BKPyVN-Mac should be interpreted as a BKPyVN-associated macrophage state that integrates viral inflammation, antigen-processing activity, and renal injury-associated remodeling, rather than as a population defined solely by proportional expansion.

GO functional enrichment analysis suggested that BKPyVN-Mac marker genes were associated with a pro-inflammatory and pro-adhesive phenotype. The enrichment of GO terms such as lymphocyte activation and cell killing suggests that BKPyVN-Mac may actively participate in the inflammatory immune microenvironment rather than serving as passive bystanders (34). Through antigen-presentation-related programs and cytokine/chemokine-associated signaling, BKPyVN-Mac may contribute to lymphocyte recruitment and activation, thereby promoting sustained inflammatory injury within the allograft (35). Additionally, enrichment of cell–substrate junction and cadherin binding GO terms suggests that BKPyVN-Mac may possess enhanced adhesive and tissue-retention properties. These adhesive features may facilitate local macrophage retention and contribute to persistent tissue injury, early remodeling, and fibrotic progression.

Pseudotime analysis positioned BKPyVN-Mac along a distinct macrophage-state transition trajectory, suggesting that this macrophage state may be influenced by the BKPyVN microenvironment. CellChat analysis provided further mechanistic insights into how BKPyVN-Mac could engage in inflammatory crosstalk. Compared with Other-Mac, BKPyVN-Mac exhibited stronger incoming and outgoing communication signals, with increased activity of ADGRE, SELL, SPP1, FN1, ANNEXIN, and ANGPTL signaling. These findings complement the interferon-responsive and antigen-processing marker profile described above and further suggest that BKPyVN-Mac may function as an active inflammatory communication hub in BKPyVN.

Among the pathways inferred by CellChat, ADGRE and SELL signaling are likely relevant to macrophage adhesion, migration, tissue retention, and immune cell recruitment, potentially sustaining local inflammatory injury within the renal allograft (36). Meanwhile, SPP1 and FN1 signaling may connect BKPyVN-Mac to extracellular matrix remodeling and fibroblast activation, linking chronic inflammation to the progression of interstitial fibrosis (32, 37).

Having characterized the intercellular communication features of BKPyVN-Mac, we employed hdWGCNA to identify co-expression gene modules (MD2, MD4, MD9, MD13, and MD14) closely associated with the BKPyVN-Mac phenotype, with the aim of exploring its intrinsic transcriptional programs. GO enrichment analysis of these modules highlighted terms related to protein synthesis, RNA binding, and RNA processing. These findings suggest that BKPyVN-Mac may possess enhanced biosynthetic activity, consistent with its inferred role as an active communication hub and immune effector population. Enrichment of apoptosis-regulation and cell-adhesion-related terms further supports the findings from our preceding single-cell and cell–cell communication analyses. This suggests that BKPyVN-Mac may maintain cell survival and tissue retention within the inflammatory microenvironment through anti-apoptotic and adhesion-related transcriptional programs.

Given that the BKPyVN-Mac-associated gene modules not only exhibit biological specificity but may also hold clinical diagnostic value, we leveraged these genes to develop a machine learning-based diagnostic model. Among nine algorithms evaluated, the Support Vector Machine (SVM) demonstrated superior performance (AUC = 0.923), indicating that the molecular signature of BKPyVN-Mac serves as a robust biomarker for distinguishing BKPyVN from STK. This signature may serve as a candidate tissue-based molecular aid for BKPyVN diagnosis; however, because it was derived from renal allograft transcriptomic data, it should not yet be regarded as an established non-invasive diagnostic or early-warning tool. Future studies should determine whether ATF4, MCL1, or related signature genes can be adapted for blood- or urine-based monitoring (38). To decode the biological basis of this diagnostic capability, we prioritized the top five genes contributing to the model—ATF4, CD63, C16orf72, GNS, and MCL1—for further analysis.

Among the top SVM-derived feature genes, ATF4 and MCL1 exhibited complementary yet distinct pathway-association profiles. ATF4, a central effector of the integrated stress response, was primarily associated with stress-response and tissue injury/remodeling-related pathways, including hypoxia, apoptosis, the p53 pathway, apical junction, and epithelial–mesenchymal transition (39–41). In contrast, MCL1, a key anti-apoptotic member of the BCL-2 family, showed broader positive associations with immune-inflammatory and survival-related pathways, including allograft rejection, inflammatory response, TNFA signaling via NF-κB, IL6–JAK–STAT3 signaling, interferon responses, complement activation, apoptosis, and PI3K–AKT–mTOR signaling (42, 43). These findings suggest that ATF4-associated stress-adaptation programs and MCL1-associated survival and inflammatory programs may collectively support the maintenance of BKPyVN-associated macrophage states within the inflammatory allograft microenvironment. However, a direct regulatory interaction between ATF4 and MCL1 was not examined in the present study and requires experimental validation. The remaining SVM-derived feature genes—CD63, C16orf72, and GNS—showed more heterogeneous pathway-association profiles. CD63 was linked to tissue-remodeling-related pathways, whereas C16orf72 and GNS were mainly associated with metabolic and protein-secretion-related programs (44–46). These findings suggest that, beyond ATF4- and MCL1-related stress and survival programs, the SVM-derived signature may also reflect vesicle/lysosome-associated activity and metabolic remodeling in BKPyVN-associated macrophage states.

Based on these findings, we propose a putative model in which persistent BKPyV replication exposes the allograft microenvironment to viral antigens, interferon-related inflammatory signals, and cellular stress cues. These sustained stimuli may promote macrophage transition toward a BKPyVN-associated state characterized by antiviral, antigen-processing, stress-adaptive, survival-related, and remodeling-associated programs. In this context, ATF4- and MCL1-associated programs may support macrophage maintenance, while ADGRE/SELL- and SPP1/FN1-related signaling may link inflammatory recruitment with extracellular matrix remodeling. Thus, ATF4 and MCL1 should be interpreted not as isolated fibrosis markers, but as components of a stress-adaptation/survival axis that may sustain BKPyVN-associated macrophage states and indirectly contribute to fibrotic progression.

To validate the bioinformatics findings in a living system, we established a persistent MPyV infection model in 2-week-old male C57BL/6 mice. MPyV is an ideal surrogate model for studying polyomavirus-associated nephropathy due to its high genomic and biological homology (>70%) with human BKPyV, as well as its similar tropism for the urinary epithelium (47, 48). It is important to note that while clinical BKPyVN typically occurs in the context of ischemia-reperfusion injury (IRI) and potent immunosuppression (IS) following kidney transplantation, we deliberately employed a simplified infection model in wild-type mice without these confounding factors. This reductionist approach was designed to isolate the primary host response and baseline expression of molecular biomarkers during natural viral colonization, free from the interference of clinical externalities. Our results demonstrate that persistent MPyV infection alone is sufficient to trigger significant localized cellular stress and inflammatory responses. In the persistent MPyV infection model, ATF4 and MCL1 showed infection-associated induction at both the protein and transcript levels, broadly consistent with the persistent renal viral burden observed during the late phase of infection. Importantly, the increased ATF4 and MCL1 mRNA expression and stronger immunohistochemical immunoreactivity observed in human BKPyVN biopsy samples further support the clinical relevance of these findings. These observations suggest that ATF4/MCL1-associated stress-response and cell-survival programs may constitute shared molecular responses involved in polyomavirus-mediated renal injury.

On the basis of this *in vivo* evidence, we further explored the predicted perturbation networks associated with ATF4 and MCL1 using scTenifoldKnk-based virtual knockout analysis. ATF4 perturbation induced broad changes involving metabolic genes, mitochondrial and oxidoreductase/NAD-related functions, and MHC class II-associated genes, including HLA-DRA, CD74, and HLA-DRB1 (49, 50). These findings suggest that ATF4 may coordinate a stress-adaptive immunometabolic program that supports amino acid utilization, redox homeostasis, mitochondrial remodeling, and biosynthetic activity under persistent viral antigen exposure and inflammatory stress, while also contributing to MHC class II-mediated antigen processing and presentation in BKPyVN-Mac (51). In contrast, MCL1 perturbation produced a more restricted network involving DUSP2, DUSP4, CXCR4, NR4A2, and CREM. As an anti-apoptotic BCL-2 family protein, MCL1 may support macrophage survival within the inflammatory allograft microenvironment, whereas DUSP2/DUSP4-mediated MAPK feedback may modulate the magnitude and duration of inflammatory signaling (52). Thus, the predicted MCL1–DUSP2/DUSP4 relationship raises a testable hypothesis that MCL1 helps balance macrophage persistence and inflammatory activation in BKPyVN-Mac.

Although this study provides novel insights into BKPyVN-associated macrophage states and potential biomarkers, several limitations should be acknowledged. First, the MPyV infection model was established in wild-type C57BL/6 mice without kidney transplantation or immunosuppressive treatment; therefore, it cannot fully recapitulate the clinical setting of post-transplant BKPyVN. This model should be interpreted as a simplified *in vivo* system for evaluating conserved host responses to persistent renal polyomavirus infection, rather than as a complete model of post-transplant BKPyVN under immunosuppression. The potential translational relevance of ATF4 and MCL1 is supported by convergent evidence from human transcriptomic analysis, increased ATF4 and MCL1 mRNA expression in human BKPyVN biopsy samples, and time-course validation in MPyV-infected kidneys showing induction and sustained elevation of ATF4/MCL1 proteins and Atf4/Mcl1 transcripts; nevertheless, further validation in transplant-based or immunosuppression-treated models is required. Second, the findings derived from virtual knockout analysis should be interpreted as computational predictions and require further validation in appropriate *in vitro* cell models. Third, this study primarily focused on the macrophage compartment. Given that BKPyVN involves dynamic interactions among multiple cell types, including tubular epithelial cells and infiltrating T cells, further investigation of multicellular crosstalk within the allograft niche is warranted to better define BKPyVN pathogenesis.

## 5 Conclusion

In conclusion, this study identified BKPyVN-Mac as a disease-associated macrophage population linked to the pro-inflammatory and pro-fibrotic microenvironment of BKPyVN based on single-cell transcriptomic analysis. By integrating hdWGCNA with machine learning, we developed an SVM-based diagnostic model with favorable performance (AUC = 0.923) and identified a core five-gene signature, among which ATF4 and MCL1 emerged as stress- and survival-associated feature genes. The relevance of ATF4 and MCL1 was further supported by human transcriptomic analysis, RT-qPCR and immunohistochemical validation in renal allograft biopsies, as well as by time-course transcript and protein analyses in a persistent MPyV infection model. These findings provide new insights into the immunometabolic and stress-adaptive programs associated with BKPyVN and support ATF4 and MCL1 as candidate diagnostic biomarkers for future validation.

## Data Availability

The raw data supporting the conclusions of this article will be made available by the authors, without undue reservation.

## References

[1] Sawinski D Goral S . BK virus infection: an update on diagnosis and treatment. Nephrol Dial Transplant. (2015) 30:209–17. doi: 10.1093/ndt/gfu023 24574543

[2] Hirsch HH Brennan DC Drachenberg CB Ginevri F Gordon J Limaye AP . Polyomavirus-associated nephropathy in renal transplantation: interdisciplinary analyses and recommendations. Transplantation. (2005) 79:1277–86. doi: 10.1097/01.tp.0000156165.83160.09 15912088

[3] Jiang M Abend JR Tsai B Imperiale MJ . Early events during BK virus entry and disassembly. J Virol. (2009) 83:1350–8. doi: 10.1128/jvi.02169-08 19036822 PMC2620883

[4] Ambalathingal GR Francis RS Smyth MJ Smith C Khanna R . BK Polyomavirus: Clinical aspects, immune regulation, and emerging therapies. Clin Microbiol Rev. (2017) 30:503–28. doi: 10.1128/cmr.00074-16 28298471 PMC5355639

[5] Li P Cheng D Wen J Ni X Li X Xie K . The immunophenotyping of different stages of BK virus allograft nephropathy. Ren Fail. (2019) 41:855–61. doi: 10.1080/0886022x.2019.1617168 31535918 PMC6758702

[6] Cui Y Chen C Tang Z Yuan W Yue K Cui P . TREM2 deficiency aggravates renal injury by promoting macrophage apoptosis and polarization via the JAK-STAT pathway in mice. Cell Death Dis. (2024) 15:401. doi: 10.1038/s41419-024-06756-w 38849370 PMC11161629

[7] Cohen C Mhaidly R Croizer H Kieffer Y Leclere R Vincent-Salomon A . WNT-dependent interaction between inflammatory fibroblasts and FOLR2+ macrophages promotes fibrosis in chronic kidney disease. Nat Commun. (2024) 15:743. doi: 10.1038/s41467-024-44886-z 38272907 PMC10810789

[8] Li L Liao J Zhang Y Yao Z Huang J Wu K . Single cell and spatial transcriptomics define a proinflammatory and profibrotic niche after kidney injury. Adv Sci (Weinh). (2025) 13:e03691. doi: 10.1002/advs.202503691 41042124 PMC12786295

[9] Lubetzky M Bao Y P OB Marfo K Ajaimy M Aljanabi A . Genomics of BK viremia in kidney transplant recipients. Transplantation. (2014) 97:451–6. doi: 10.1097/01.tp.0000437432.35227.3e 24310299

[10] Sigdel TK Bestard O Salomonis N Hsieh SC Torras J Naesens M . Intragraft antiviral-specific gene expression as a distinctive transcriptional signature for studies in polyomavirus-associated nephropathy. Transplantation. (2016) 100:2062–70. doi: 10.1097/tp.0000000000001214 27140517 PMC5235336

[11] Sigdel TK Gao Y He J Wang A Nicora CD Fillmore TL . Mining the human urine proteome for monitoring renal transplant injury. Kidney Int. (2016) 89:1244–52. doi: 10.1016/j.kint.2015.12.049 27165815 PMC5221536

[12] Leek JT Johnson WE Parker HS Jaffe AE Storey JD . The sva package for removing batch effects and other unwanted variation in high-throughput experiments. Bioinformatics. (2012) 28:882–3. doi: 10.1093/bioinformatics/bts034 22257669 PMC3307112

[13] Hao Y Stuart T Kowalski MH Choudhary S Hoffman P Hartman A . Dictionary learning for integrative, multimodal and scalable single-cell analysis. Nat Biotechnol. (2024) 42:293–304. doi: 10.1038/s41587-023-01767-y PMC10928517 37231261

[14] McGinnis CS Murrow LM Gartner ZJ . DoubletFinder: Doublet detection in single-cell RNA sequencing data using artificial nearest neighbors. Cell Syst. (2019) 8:329–337.e4. doi: 10.1016/j.cels.2019.03.003 30954475 PMC6853612

[15] Korsunsky I Millard N Fan J Slowikowski K Zhang F Wei K . Fast, sensitive and accurate integration of single-cell data with Harmony. Nat Methods. (2019) 16:1289–96. doi: 10.1038/s41592-019-0619-0 31740819 PMC6884693

[16] Trapnell C Cacchiarelli D Grimsby J Pokharel P Li S Morse M . The dynamics and regulators of cell fate decisions are revealed by pseudotemporal ordering of single cells. Nat Biotechnol. (2014) 32:381–6. doi: 10.1038/nbt.2859 24658644 PMC4122333

[17] Qiu X Hill A Packer J Lin D Ma YA Trapnell C . Single-cell mRNA quantification and differential analysis with Census. Nat Methods. (2017) 14:309–15. doi: 10.1038/nmeth.4150 28114287 PMC5330805

[18] Jin S Plikus MV Nie Q . CellChat for systematic analysis of cell-cell communication from single-cell transcriptomics. Nat Protoc. (2025) 20:180–219. doi: 10.1038/s41596-024-01045-4 39289562

[19] Morabito S Reese F Rahimzadeh N Miyoshi E Swarup V . hdWGCNA identifies co-expression networks in high-dimensional transcriptomics data. Cell Rep Methods. (2023) 3:100498. doi: 10.1016/j.crmeth.2023.100498 37426759 PMC10326379

[20] Sanz H Valim C Vegas E Oller JM Reverter F . SVM-RFE: selection and visualization of the most relevant features through non-linear kernels. BMC Bioinf. (2018) 19:432. doi: 10.1186/s12859-018-2451-4 30453885 PMC6245920

[21] Boulesteix AL Strimmer K . Partial least squares: a versatile tool for the analysis of high-dimensional genomic data. Brief Bioinform. (2007) 8:32–44. doi: 10.1093/bib/bbl016 16772269

[22] Luna JM Gennatas ED Ungar LH Eaton E Diffenderfer ES Jensen ST . Building more accurate decision trees with the additive tree. Proc Natl Acad Sci USA. (2019) 116:19887–93. doi: 10.1073/pnas.1816748116 31527280 PMC6778203

[23] Dou B Zhu Z Merkurjev E Ke L Chen L Jiang J . Machine learning methods for small data challenges in molecular science. Chem Rev. (2023) 123:8736–80. doi: 10.1021/acs.chemrev.3c00189 37384816 PMC10999174

[24] Newman AM Liu CL Green MR Gentles AJ Feng W Xu Y . Robust enumeration of cell subsets from tissue expression profiles. Nat Methods. (2015) 12:453–7. doi: 10.1038/nmeth.3337 25822800 PMC4739640

[25] Osorio D Zhong Y Li G Xu Q Yang Y Tian Y . scTenifoldKnk: An efficient virtual knockout tool for gene function predictions via single-cell gene regulatory network perturbation. Patterns (N Y). (2022) 3:100434. doi: 10.1016/j.patter.2022.100434 35510185 PMC9058914

[26] Huang G Zeng G Huang Y Ramaswami B Randhawa P . Evaluation of the gastrointestinal tract as potential route of primary polyomavirus infection in mice. PloS One. (2016) 11:e0150786. doi: 10.1371/journal.pone.0150786 26939117 PMC4777556

[27] Kotton CN Kamar N Wojciechowski D Eder M Hopfer H Randhawa P . The second international consensus guidelines on the management of BK polyomavirus in kidney transplantation. Transplantation. (2024) 108:1834–66. doi: 10.1097/tp.0000000000004976 38605438 PMC11335089

[28] Tang Y Wang Z Du D . Challenges and opportunities in research on BK virus infection after renal transplantation. Int Immunopharmacol. (2024) 141:112793. doi: 10.1016/j.intimp.2024.112793 39146777

[29] Udomkarnjananun S Iampenkhae K . Pathological approach to kidney allograft infection. Biomedicines. (2023) 11. doi: 10.3390/biomedicines11071902 37509541 PMC10377023

[30] Conway BR O'Sullivan ED Cairns C O'Sullivan J Simpson DJ Salzano A . Kidney single-cell atlas reveals myeloid heterogeneity in progression and regression of kidney disease. J Am Soc Nephrol. (2020) 31:2833–54. doi: 10.1681/asn.2020060806 32978267 PMC7790206

[31] Yao W Chen Y Li Z Ji J You A Jin S . Single cell RNA sequencing identifies a unique inflammatory macrophage subset as a druggable target for alleviating acute kidney injury. Adv Sci (Weinh). (2022) 9:e2103675. doi: 10.1002/advs.202103675 35112806 PMC9036000

[32] Hoeft K Schaefer GJL Kim H Schumacher D Bleckwehl T Long Q . Platelet-instructed SPP1(+) macrophages drive myofibroblast activation in fibrosis in a CXCL4-dependent manner. Cell Rep. (2023) 42:112131. doi: 10.1016/j.celrep.2023.112131 36807143 PMC9992450

[33] Zhang YL Tang TT Wang B Wen Y Feng Y Yin Q . Identification of a novel ECM remodeling macrophage subset in AKI to CKD transition by integrative spatial and single-cell analysis. Adv Sci (Weinh). (2024) 11:e2309752. doi: 10.1002/advs.202309752 39119903 PMC11481374

[34] Rowshani AT Vereyken EJ . The role of macrophage lineage cells in kidney graft rejection and survival. Transplantation. (2012) 94:309–18. doi: 10.1097/tp.0b013e318250c10f 22828735

[35] Panzer SE . Macrophages in transplantation: a matter of plasticity, polarization, and diversity. Transplantation. (2022) 106:257–67. doi: 10.1097/TP.0000000000003804 PMC8548398 33908380

[36] Wang S Sun Z Zhao W Wang Z Wu M Pan Y . CD97/ADGRE5 inhibits LPS induced NF-κB activation through PPAR-γ upregulation in macrophages. Mediators Inflammation. (2016) 2016:1605948. doi: 10.1155/2016/1605948 PMC4779537 26997758

[37] Vegting Y Jongejan A Neele AE Claessen N Sela G Prange KHM . Infiltrative classical monocyte-derived and SPP1 lipid-associated macrophages mediate inflammation and fibrosis in ANCA-associated glomerulonephritis. Nephrol Dial Transplant. (2025) 40:1416–27. doi: 10.1093/ndt/gfae292 39694817

[38] Zhang Z Cao C Garcia Haider C Wang Y Zhu Y Abdelhak O . Diagnostic value of three urine miRNAs composite score in BK virus nephropathy. Clin Chim Acta. (2025) 569:120162. doi: 10.1016/j.cca.2025.120162 39870292

[39] van der Mijn JC Chen Q Laursen KB Khani F Wang X Dorsaint P . Transcriptional and metabolic remodeling in clear cell renal cell carcinoma caused by ATF4 activation and the integrated stress response (ISR). Mol Carcinog. (2022) 61:851–64. doi: 10.1002/mc.23437 35726553 PMC9378514

[40] Labbé K LeBon L King B Vu N Stoops EH Ly N . Specific activation of the integrated stress response uncovers regulation of central carbon metabolism and lipid droplet biogenesis. Nat Commun. (2024) 15:8301. doi: 10.1038/s41467-024-52538-5 PMC11436933 39333061

[41] Sanfrancesco VC Hood DA . Acute contractile activity induces the activation of the mitochondrial integrated stress response and the transcription factor ATF4. J Appl Physiol (1985). (2025) 138:857–71. doi: 10.1152/japplphysiol.00307.2024 39417830

[42] Widden H Placzek WJ . The multiple mechanisms of MCL1 in the regulation of cell fate. Commun Biol. (2021) 4:1029. doi: 10.1038/s42003-021-02564-6 34475520 PMC8413315

[43] Okamoto T Coultas L Metcalf D van Delft MF Glaser SP Takiguchi M . Enhanced stability of Mcl1, a prosurvival Bcl2 relative, blunts stress-induced apoptosis, causes male sterility, and promotes tumorigenesis. Proc Natl Acad Sci USA. (2014) 111:261–6. doi: 10.1073/pnas.1321259110 PMC3890801 24363325

[44] Li S Li M Zhang Y Chen J Li J Fu S . The role of tetraspanins in organ fibrosis: mechanisms and therapeutic perspectives. J Transl Med. (2025) 23:1007. doi: 10.1186/s12967-025-06890-9 40993816 PMC12462079

[45] Amici DR Ansel DJ Metz KA Smith RS Phoumyvong CM Gayatri S . C16orf72/HAPSTR1 is a molecular rheostat in an integrated network of stress response pathways. Proc Natl Acad Sci USA. (2022) 119:e2111262119. doi: 10.1073/pnas.2111262119 35776542 PMC9271168

[46] Benslimane Y Sánchez-Osuna M Coulombe-Huntington J Bertomeu T Henry D Huard C . A novel p53 regulator, C16ORF72/TAPR1, buffers against telomerase inhibition. Aging Cell. (2021) 20:e13331. doi: 10.1111/acel.13331 33660365 PMC8045932

[47] Albrecht JA Dong Y Wang J Breeden C Farris AB 3rd . Adaptive immunity rather than viral cytopathology mediates polyomavirus-associated nephropathy in mice. Am J Transplant. (2012) 12:1419–28. doi: 10.1111/j.1600-6143.2012.04005.x 22420885 PMC3365603

[48] Chen XT Huang Y Wang J Li G Zhang Y He LF . Ischemia-reperfusion injury and immunosuppressants promote polyomavirus replication through common molecular mechanisms. Front Immunol. (2022) 13:835584. doi: 10.3389/fimmu.2022.835584 35281039 PMC8914341

[49] Pandey J Larson-Casey JL Patil MH He C Pinthong N Carter AB . The PERK/ATF4 pathway is required for metabolic reprogramming and progressive lung fibrosis. JCI Insight. (2025) 10. doi: 10.1172/jci.insight.189330 40208691 PMC12128959

[50] Yan X Liu C . The ATF4-glutamine axis: a central node in cancer metabolism, stress adaptation, and therapeutic targeting. Cell Death Discov. (2025) 11:390. doi: 10.1038/s41420-025-02683-7 40830338 PMC12365302

[51] Wang C Chen Q Chen S Fan L Gan Z Zhao M . Serine synthesis sustains macrophage IL-1β production via NAD(+)-dependent protein acetylation. Mol Cell. (2024) 84:744–759.e6. doi: 10.1016/j.molcel.2024.01.002 38266638

[52] Zhang C Zhao S Tan Y Pan S An W Chen Q . The SKA3-DUSP2 axis promotes gastric cancer tumorigenesis and epithelial-mesenchymal transition by activating the MAPK/ERK pathway. Front Pharmacol. (2022) 13:777612. doi: 10.3389/fphar.2022.777612 35295342 PMC8918524

