# Supplementary material for: BKPyVN-associated macrophage subpopulations and diagnostic biomarker identification - combine machine learning with single-cell analysis
Source: Front Immunol. 2026 Oct 14;17:1881621. doi: 10.3389/fimmu.2026.1881621 (PMC13713854; doi:10.3389/fimmu.2026.1881621)
Supplement: Supplementary file 1 [file SupplementaryFile1.docx]

**Figure S1.** Differential inferred signaling landscape of intercellular communication pathways across cell types in BKPyVN compared with STK. (**A**) Heatmap showing differences in inferred incoming signaling patterns received by distinct cell populations. (**B**) Heatmap showing differences in inferred outgoing signaling patterns originating from distinct cell populations.

**Figure S2.** Co-expression networks of hub genes in BKPyVN-Mac-associated modules. (**A–E**) Network visualizations of the top hub genes within the five modules most strongly associated with the BKPyVN-Mac phenotype: MD2, MD4, MD9, MD13, and MD14. Nodes represent hub genes selected based on module eigengene-based connectivity (kME), whereas edges indicate inferred co-expression relationships.

**Figure S3.** ROC analysis of candidate diagnostic genes. (**A–M**) ROC curves for candidate genes derived from the key co-expression modules. Only genes with an AUC > 0.80 are displayed, suggesting their potential diagnostic value for distinguishing BKPyVN from STK. The corresponding AUC value is indicated in each panel.

**Figure S4.** Characteristics of machine learning-identified feature genes and correlations among CIBERSORT-derived immune cell fractions. (**A**) Visualization of the chromosomal locations of the top five feature genes identified by the SVM model: ATF4, CD63, C16orf72, GNS, and MCL1. (**B–I**) The top 10 feature genes ranked by importance across the eight remaining machine learning algorithms: glmBoost, KNN, NeuralNet, PLS, Logistic Regression, XGBoost, GBM, and DTS. (**J**) Correlation matrix heatmap showing pairwise correlations among CIBERSORT-derived immune cell fractions. The color scale indicates the strength and direction of each pairwise correlation.

**Figure S5.** Exploratory correlations between key feature genes and CIBERSORT-estimated immune-cell fractions. (**A–C**) Spearman correlations between ATF4 expression and the estimated fractions of activated dendritic cells (**A**), resting dendritic cells (**B**), and M1 macrophages (**C**). (**D–E**) Spearman correlations between C16orf72 expression and M1 macrophages (**D**) and monocytes (**E**). (**F–H**) Spearman correlations between CD63 expression and M1 macrophages (**F**), M2 macrophages (**G**), and naive CD4+ T cells (**H**). (**I–J**) Spearman correlations between MCL1 expression and monocytes (**I**) and γδ T cells (**J**). Spearman’s correlation coefficient and nominal, unadjusted P value are shown in each panel. None of these correlations remained statistically significant after Benjamini–Hochberg false discovery rate correction; therefore, they are presented as exploratory associations.

**Figure S6.** Individual regression plots of significant ATF4/MCL1-related pathways in BKPyVN samples. Each dot represents one BKPyVN sample. The x-axis shows ATF4 or MCL1 expression, and the y-axis shows the corresponding ssGSEA pathway score. The fitted line and shaded area indicate the linear regression fit and 95% confidence interval. Spearman’s rho and cor FDR indicate the Spearman correlation coefficient and Benjamini–Hochberg-adjusted P value, respectively. Beta, 95% CI, R², and lm FDR indicate the standardized regression coefficient, its 95% confidence interval, model R², and FDR-adjusted P value for the linear regression, respectively.
